# Impact of Fixed Oil on Ostwald Ripening of Anti-Oral Cancer Nanoemulsions Loaded with *Amomum kravanh* Essential Oil

**DOI:** 10.3390/pharmaceutics14050938

**Published:** 2022-04-26

**Authors:** Yotsanan Weerapol, Suwisit Manmuan, Nattaya Chaothanaphat, Siriporn Okonogi, Chutima Limmatvapirat, Sontaya Limmatvapirat, Sukannika Tubtimsri

**Affiliations:** 1 Faculty of Pharmaceutical Sciences, Burapha University, Chonburi 20131, Thailand; yotsanan@go.buu.ac.th (Y.W.); suwisit@go.buu.ac.th (S.M.); nattaya.chao@gmail.com (N.C.); 2 Research Center of Pharmaceutical Nanotechnology, Faculty of Pharmacy, Chiang Mai University, Chiang Mai 50200, Thailand; okng2000@gmail.com; 3 Department of Pharmaceutical Sciences, Faculty of Pharmacy, Chiang Mai University, Chiang Mai 50200, Thailand; 4 Department of Pharmaceutical Chemistry, Faculty of Pharmacy, Silpakorn University, Nakhon Pathom 73000, Thailand; limmatvapirat_c@su.ac.th; 5 Department of Pharmaceutical Technology, Faculty of Pharmacy, Silpakorn University, Nakhon Pathom 73000, Thailand; limmatvapirat_s@su.ac.th

**Keywords:** amomum kravanh, essential oil, ostwald ripening, nanoemulsions, oral cancer, apoptosis

## Abstract

Recently, essential oil from *Amomum kravanh* (AMO) was reported to exert anti-oral cancer effects. Although it was more effective after being loaded into nanoemulsions, AMO without an Ostwald ripening inhibitor was unable to form stable nanoemulsions because of the Ostwald ripening phenomenon. In this study, we examined the influence of Ostwald ripening inhibitors, such as fixed oils and polyethylene glycol 4000 (PEG 4000), on nanoemulsion properties prepared by a phase inversion temperature method. Several fixed oils, including virgin coconut oil (VCO), palm oil (PMO), olive oil (OLO), and PEG 4000, were evaluated, and their Ostwald ripening inhibitory effects were compared. The results suggest that the type and ratio of AMO:fixed oils influence the formation and characteristics of nanoemulsions. PEG 4000 was unable to produce nanoemulsions; however, stable nanoemulsions with small droplet sizes were observed in preparations containing OLO and VCO at an AMO:fixed oil ratio of 80:20, which may be the result of specific molecular interactions among the components. Using an MTT assay, we demonstrated that the AMO:OLO (80:20) nanoemulsion produced the most significant cytotoxic effect on oral cancer cells with a percentage of 99.68 ± 0.56%. Furthermore, the AMO:OLO 80:20 nanoemulsion inhibits metastasis and induces oral cancer cell death through the intrinsic apoptosis pathway. In conclusion, AMO nanoemulsion with anti-oral cancer activity was successfully produced by varying the amount and type of fixed oils. In the future, this discovery may lead to the development of stable nanoemulsions employing additional volatile oils.

## 1. Introduction

*Amomum kravanh*, which belongs to the Zingiberaceae family, is a plant that is primarily found in Vietnam, China, and Thailand. It is used as a folk medicine for the treatment of digestive disorders and stomach diseases [1]. Recently, *Amomum kravanh* essential oil (AMO) has been shown to exhibit cytotoxic effects on various cancer cells in vitro, such as prostate, skin [2], and oral cancer cells [3]. Unfortunately, its use in native form results in a low bioavailability because of its low water solubility. To overcome this problem, nanoemulsions have been used as a carrier to deliver AMO more effectively [3].

Nanoemulsions represent a colloid system consisting of water and oil, stabilized by an emulsifier [4]. They are often used for substances with low water solubility because of their effective drug delivery capability [5]. Several studies have demonstrated the advantages of using nanoemulsions as carriers for anticancer agents, especially in topical dosage form. Cancer treatments using topical dosage forms are becoming a trend, especially for cancers occurring on the surface of the body, such as skin or oral cancer. This not only improves patient compliance but reduces serious systemic side effects. Regarding efficacy, anticancer agents remain highly effective after loading into topical nanoemulsions [6].

Low- and high-energy techniques can be used to prepare nanoemulsions containing essential oils [7], whereas low-energy methods are used more often for nanoemulsion preparation because this approach does not require large energy produced from expensive tools [8]. The phase inversion temperature (PIT) method is classified as a low-energy method for the preparation of nanoemulsions by varying the solubility of surfactants according to temperature change [9,10]. Several reports describe the successful preparation of nanoemulsions containing essential oils using the PIT method. Chang et al. succeeded in preparing nanoemulsions loaded with orange oil by the PIT method with various types and amounts of Tween and carrier oils (medium chain triglyceride) [11]. Similarly, peppermint oil-loaded nanoemulsions have been prepared by the addition of medium chain triglyceride and succinylated waxy maize starch as a carrier oil and emulsifier, respectively [12]. Chuesiang et al. demonstrated the feasibility of preparing nanosized and highly stable cinnamon oil nanoemulsions by incorporating 60% medium chain triglyceride into the oil phase [13].

One of the major problems encountered with the application of nanoemulsions as carriers for essential oils is the instability of emulsions (e.g., creaming and cracking). They may undergo a variety of destabilizing events upon storage, in particular, Ostwald ripening [14]. With respect to the composition of the oil phase in a previous report [3], AMO without fixed oil cannot be formed into stable nanoemulsions. Ostwald ripening is a significant destabilizing phenomenon of nanoemulsions, which leads to irreversible phase separation. This process is associated with the diffusive migration of essential oil from smaller to larger droplets as evidenced by droplet diameter growth over time. It is driven by a variety of factors according to the Lifshitz–Slyozov–Wagner theory as presented in the equation [15]:

$$\omega_{T}=\frac{d{\bar{r}}^{3}}{dt}=\frac{8\gamma C_{w}{}^{eq}D_{w}V_{m}}{9kT}$$

where $\bar{r}$ is the mean droplet radius, *γ* is the interfacial tension between the oil and aqueous phase at the droplet surface, $V_{m}$ denotes the molecular volume of the oil, $C_{w}{}^{eq}$ represents the aqueous oil solubility, $D_{w}$ indicates the diffusivity of the oil molecule, *k* is the Boltzmann constant, and *T* is the absolute temperature.

Previous studies have revealed strategies for delaying emulsion instability caused by the Ostwald ripening phenomenon. The physical crosslinked biopolymer on the droplet surface arrests oil migration into a continuous phase, which inhibits the progression of Ostwald ripening [16]. Increases in continuous phase viscosity were found to be effective in delaying the Ostwald ripening process. Crosslinking proteins, such as sodium caseinate and transglutaminase, at the oil–water interface prior to the emulsification process significantly reduced the ripening rate of alkane nanoemulsions during extended storage periods. Moreover, the incorporation of hydrophobic compounds with poor water solubility, such as Aerosil 200 [15], fixed oil [17,18], or rosin gum [15], in the internal phase delays Ostwald ripening [11,19]. Nonetheless, few studies have demonstrated the efficacy of Ostwald ripening inhibitors, such as fixed oil and polyethylene glycol (PEG 4000), on the formation and properties of nanoemulsions, particularly AMO nanoemulsions, using a PIT method.

Despite the fact that earlier studies have shown that AMO nanoemulsions with soybean oil can serve as a stable anti-oral cancer nanoemulsion, the mechanism for nanoemulsion stabilization and anticancer activity has not been defined. Therefore, the objective of this study was to compare the performance of Ostwald ripening inhibitors, including fixed oil and PEG 4000, based on their size, polydispersity index (pDI), stability, morphology, and interactions with molecules by 2D-NOESY nuclear magnetic resonance (NMR) spectroscopy. In addition, the impact of the structure and conformation of fixed oils, including virgin coconut oil (C12, VCO), palm oil (C16, PMO), and olive oil (C18:1, OLO), were evaluated. The anticancer activity, permeability of cells, cell invasion, and cell death mechanisms induced by AMO nanoemulations were also determined.

## 2. Method

### 2.1. Materials

Fruits of *Amomum kravanh* were collected from plants harvested in Chanthaburi, Thailand. Its essential oil was produced by hydro-distillation according to a previously described method [3]. A human oral squamous cancer cell line (KON; Lot No. 01262007) was obtained from the JCRB cell bank, Japan. Other chemicals use for the experiments were as follows: 3-(4,5-dimethylthiazol-2-yl)-2,5-diphenyltetrazolium bromide (MTT, Lot No. 39H5076, Sigma-Aldrich, St Louis, MO, USA), Kolliphor EL (Lot No. 04321136W0, BASF, Ludwigshafen, Germany), VCO (Lot No. SS54/06177−01, Tropicana Oil, Nakhon Pathom, Thailand), PMO (Lot No. B029P6, Morakot Industries, Bangkok, Thailand), OLO (Lot No. L72039B-28776, P. C. Drug Center, Bangkok, Thailand), and PEG 4000 (Lot No. 1304139947, Ajax Finechem Pty, Sydney, Australia).

### 2.2. Nanoemulsion Preparation

The PIT approach was used to prepare AMO:fixed oil nanoemulsions. The oil (AMO:fixed oil in a total of 10% *w*/*w* and 10% *w*/*w* of Kolliphor EL) and water phases were heated to 62 and 65 °C, respectively. After reaching the specified temperature, a homogenizer (IKA T25 digital, Staufen, Germany) was used to blend the oil and water phases for 5 min at 3800 rpm. The effect of AMO:fixed oil ratios (0:100, 30:70, 50:50, 70:30, 80:20, 90:10 and 100:0) and the type of fixed oil (VCO, PMO, OLO) on the characteristics of the corresponding nanoemulsions were evaluated. Additionally, PEG 4000 was added to some formulations to determine the inhibitory effect on Ostwald ripening. The composition of each nanoemulsion is presented in Table 1.

### 2.3. Droplet Size and Size Distribution

Dynamic light scattering (Zetasizer Nano-ZS, Malvern Instruments, Worcestershire, UK) with a capability of measuring droplet size in a range of 0.3 nm–10 µm was used to determine the droplet size and polydispersity index of AMO nanoemulsions. The experiments were run in triplicate and recorded as the mean ± SD.

### 2.4. Oil Droplet Morphology

The morphology of oil droplets was examined using optical microscopy and atomic force microscopy (AFM). The optical microscope (CX41 RF, Olympus, Shibuya-ku, Japan) was used to evaluate the emulsions with micron-sized droplets, whereas AFM was used to assess nanosized emulsions (Nanowizard III, JPK, Berlin, Germany). Each sample was directly placed onto the slide and viewed with the aid of a digital eye piece (AM423X, ANMO Electronic, New Taipei City, Taiwan). AFM was used to examine emulsions containing submicron droplets, particularly those less than 100 nm in diameter. All materials were diluted 10-fold before depositing onto a freshly prepared mica disk and dried at room temperature in a fume hood. The dried samples were analyzed using the nano-probe cantilever’s tapping mode.

### 2.5. Stability

A temperature cycling test was performed to assess the stability of the AMO nanoemulsion. Three bottles of each sample were incubated at 45 or 4 °C for 24 h, equaling 1 cycle, for a total of 6 cycles. Afterward, the nanoemulsions were evaluated for their properties relative to that of the initial values.

### 2.6. ^1^H–^1^H (NOESY) NMR Spectroscopy

The interaction among components in nanoemulsion systems was investigated using 2D-NOESY NMR spectroscopy. The nanoemulsion was produced as described in nanoemulsion preparation, except that deuterium oxide (D_2_O) was used instead of water. Each sample was then analyzed using a Varian 400 MHz NMR spectroscopy (AVANCE, III HD, Bruker, Rheinstetten, Germany).

### 2.7. Transepithelial Electrical Resistance (TEER) Measurements

Enhancing permeability is an important feature of nanoemulsions, especially when using in a topical dosage formulation. For this experiment, a TEER value was used to assess the permeability of the nanoemulsions across an oral tissue barrier by the paracellular pathway. A section of porcine buccal from a local slaughterhouse was cut from the underlying tissues using surgical scissors and gently cleaned with phosphate buffer saline (PBS) prior to the test. A representative nanoemulsion was selected to compare with their corresponding solution and control (PBS). The experiment was performed using 6 hanging insert wells with a penetration area of 1.13 cm^2^. The buccal tissue was placed between the donor and receiver compartments of the diffusion cells. The donor and receiver compartments were filled with 200 and 1500 µL of PBS, respectively. A Millicell^®^ ERS-2 Voltohm meter (Merck KGaA, Darmstadt, Germany) was used to measure the TEER values. After measurement, PBS in the donor compartment was replaced by each nanoemulsion formulation in a volume of 200 µL. After 3 h, the nanoemulsion was discarded and reconstituted with 200 µL of PBS. TEER values were calculated and compared to those obtained before treatment. The TEER value was calculated as follows:

R = (R_sample_ − R_blank_)SA

where R_sample_ represents the electrical resistance value of the treated tissue (Ω), R_blank_ is the electrical resistance value of the hanging insert plates (Ω), and SA represents the surface area of the hanging insert plate membrane (cm^2^).

### 2.8. Anticancer Activity

The MTT analysis was conducted to detect cell viability. Oral cancer cells (KON) and normal fibroblast cells (MRC-5) were individually cultured prior to the test using Dulbecco’s modified eagle medium (DMEM) supplemented with 10% fetal bovine serum (FBS) and 0.01% of glutamine. After reaching 80% confluence, 1 × 10^4^–5 × 10^5^ KON cells and MRC-5 cells were separately seeded into a 96-well plate and cultured for 24 h at 37 °C in a 5% CO_2_ incubator (KBF-240, Binder, Tuttlingen, Germany), followed by the addition of nanoemulsions containing different fixed oils (80:20) at 0.0625–4% (*v*/*v*). After 24 h, the medium was withdrawn, and 50 µL of MTT solution (0.5 mg/mL) was added to each well and incubated under dark conditions for 3 h. Finally, 50 µL of dimethyl sulfoxide (DMSO) and the absorbance of each well was measured at 570 nm (FLUOstar Omega, BMG Labtech, Ortenberg, Germany). The number of viable cells from three experiments was determined compared with the controls (10% *w*/*w* of Kolliphor EL) and correlation between percentage of cell viability and nanoemulsion concentration were fabricated to provide IC_60_ (the concentration of nanoemulsions which demonstrated 60% cell viability) using the equation:

$$\mathrm{Cell}\;\mathrm{viability}\;(\%)=100\;\times \;\frac{\mathrm{Mean}\;\mathrm{absorbance}\;\mathrm{of}\;\mathrm{treated}\;\mathrm{cell}\;}{\mathrm{Mean}\;\mathrm{absorbance}\;\mathrm{of}\;\mathrm{untreated}\;\mathrm{cell}}$$

### 2.9. Cell Metastasis

The influence of the AMO nanoemulsion on cancer metastasis was evaluated using a cell invasion assay. Diluted Matrigel (40 µL) was added to a 24-well plate and allowed to polymerize for 24 h in a laminar hood. Next, 1 × 10^3^ KON cells were seeded into the upper compartment of the system (pore size, 8 μm), which contained the control (DMEM), AMO solution (at concentrations equivalent to that used in the nanoemulsions), representative nanoemulsions at an IC_60_ concentration, and 30 µg/mL of 5-fluorouracil (5-FU; positive control), whereas 500 µL of DMEM supplemented with 10% FBS was added to the lower chamber and incubated at 37 °C in 5% CO_2_ for 48 h. The medium was removed from both compartments. Cells that invaded through the Matrigel layer were fixed with 1 mL of ice-cold methanol at room temperature for 15 min and removed from both the upper and lower compartments. Non-invading cells were removed from the upper surface of the invasion compartment with a cotton swab. The invading cells were stained with 500 μL of crystal violet for 30 min and washed three times with PBS until the bottom of the chamber was clear. The KON cells from three experiments were counted under a 10× inverted microscope (Olympus, Tokyo, Japan) and the percentage of proportional invasiveness was calculated as followed:

$$\mathrm{Proportional}\;\mathrm{invasiveness}\;(\%)=100\;\times \;\frac{\mathrm{number}\;\mathrm{of}\;\mathrm{cells}\;\mathrm{invaded}\;\mathrm{in}\;\mathrm{treated}\;\mathrm{cells}}{\mathrm{number}\;\mathrm{of}\;\mathrm{cell}\;\mathrm{invaded}\;\mathrm{in}\;\mathrm{untreated}\;\mathrm{cells}\;}$$

### 2.10. Apoptosis Determination

Nuclear fragmentation is a hallmark feature of apoptosis. To assess nuclear fragmentation following nanoemulsion treatment, cells were examined with the fluorescent dye, 4,6-diamidino-2-phenylindole dihydrochloride (DAPI). Prior to test, KON cells were grown using DMEM supplemented with 10% FBS and 0.01% of glutamine until 80% confluent. KON cell suspensions (1 × 10^6^ cells/well) were seeded into 6-well plates for 24 h on sterile glass coverslips. Each well included the following samples:control (DMEM), base, AMO solution (at concentrations equivalent to that used in the nanoemulsions), representative nanoemulsions at the IC_60_ concentration, and 30 µg/mL of 5-FU. After 24 h, the cells were rinsed with PBS, fixed with 4% paraformaldehyde for 10 min, and permeabilized for 5 min with 0.2% Triton X−100. The fixed cells were stained with 300 mM of DAPI for 10 min and scanned with a blue filter using fluorescence microscopy (ECLIPSR Ts2, Nikon, Tokyo, Japan). The apoptotic cells of each treatment from three experiments were calculated according to the equation:

$$\mathrm{Apoptotic}\;\mathrm{cells}\;(\mathrm{fold}\;\mathrm{change})=\frac{\mathrm{Percentage}\;\mathrm{of}\;\mathrm{apoptotic}\;\mathrm{cells}\;\mathrm{of}\;\mathrm{treatment}\;\mathrm{group}\;}{\mathrm{Percentage}\;\mathrm{of}\;\mathrm{apoptotic}\;\mathrm{cells}\;\mathrm{of}\;\mathrm{untreatment}\;\mathrm{group}}$$

Phosphatidylserine exposure on cell surface was a characteristic feature of apoptosis which had specific binding with annexin V. To identify cells with apoptosis, annexin V bound to fluorescein isothiocyanate (FITC) was selected to stain cells after induction with samples. Furthermore, nuclear staining fluorescent dye, DAPI, was combined to detect necrotic or late apoptotic cells based on the loss of plasma and nuclear membrane integrity. In 6-well plate with glass coverslips, KONs were added at a density of 1 × 10^6^ cells/well for 24 h. The cells on glass coverslips were stained with annexin V-FITC/DAPI (BD Biosciences, San Diego, CA, USA) after being treated with control (DMEM), base, AMO solution (at concentrations equivalent to that used in the nanoemulsions), representative nanoemulsions at the IC_60_ concentration, and 30 µg/mL of 5-FU for 16–20 h. The labeled cells were incubated for 10 min at room temperature in the dark condition and washed with PBS before being analyzed on a fluorescent microscope (ECLIPSR Ts2, Nikon, Tokyo, Japan). Apoptotic was stained with only annexin V, while late apoptotic or necrosis was defined by staining with both of annexin V and DAPI. The number of apoptotic cells from three experiments, represented by green staining under fluorescent microscope, in each treatment was determined using the same calculation that was used in the nuclear fragmentation experiment.

Apoptosis is distinguished by the expression of apoptosis-related genes, such as Bax and Bcl-2. Apoptotic cells exhibit an increase in Bax expression (proapoptotic) and a decrease in Bcl-2 expression (anti-apoptotic). Changes in Bax and Bcl-2 expression compared with 18SrRNA (reference gene) were measured by quantitative real-time polymerase chain reaction (qPCR). KON cells were cultured by DMEM supplemented with 10% FBS and 0.01% of glutamine until 80% confluent. KON cell suspensions (1–5 × 10^6^ cells/well) were seeded into 6-well plates for 24 h. Subsequently, each well was treated with control (DMEM), base, AMO solution (at concentrations equivalent to that used in the nanoemulsions), representative nanoemulsions at the IC_60_ concentration, and 30 µg/mL of 5-FU. Total RNA was extracted using NucleoSpin^®^miRNA from KON cells after a 24 h treatment with each sample and was reverse transcribed using ReverTra Ace^®^ with oligo dT and random primers. Quantitative PCR was performed using the THUNDERBIRD^®^ SYBR qPCR mix in a 20 µL reaction volume using a quick, high-throughput, plate-based qPCR amplification method and detection apparatus (LightCycler^®^ 480 Instrument II, Roche, Switzerland). The reaction conditions were 95 °C for 45 s followed by 40 cycles of 15 s at 95 °C and 30 s at 60 °C. The primer sequences for Bax were 5′-GAGCAGATCATGAAGACAGG-3′ (forward) and 5′-GGCGGCAATCATCCTCTG-3′ (reverse); for Bcl-2, 5′-GACTTCCCGCCGCTACC-3′ (forward) and 5′-CTTCACTTGTGGCCCAGATAGG-3′ (reverse); and for 18 s rRNA, 5′-AATCAGGGTTCGATTCCGGA-3′ (forward) and 5′-CCAAGATCCAACTACGAGCT-3′ (reverse). The relative mRNA level was calculated as follows (*n* = 3) [17]:

ΔCt = Ct_target_ − Ct_ref_

ΔΔCt = ΔCt_treatment_ − ΔCt_untreated_

Relative gene expression level (fold change) = 2^−^^ΔΔCt^

Ct denotes the threshold cycle or the number of cycles required to generate fluorescence. Ct_target_ is the target gene threshold cycle (Bax or Bcl-2). Ct_ref_ indicates the threshold cycle of the reference gene (18S). ΔCt treatment is the difference in the threshold cycle between target and reference genes after treatment with base, AMO solution, AMO nanoemulsion or 30 µg/mL of 5-FU. ΔCt untreated is the difference in the threshold cycles of the target of the untreated group and reference genes.

### 2.11. Statistical Analysis

The statistical analysis was conducted using a SPSS 10.0 for Windows (SPSS Inc., Chicago, IL, USA). The data were analyzed using a paired t test and one-way ANOVA followed by Tukey’s test. Statistical significance was determined by a *p* value of less than 0.05.

## 3. Results and Discussion

### 3.1. Droplet Size and Size Distribution

**Figure 1:**
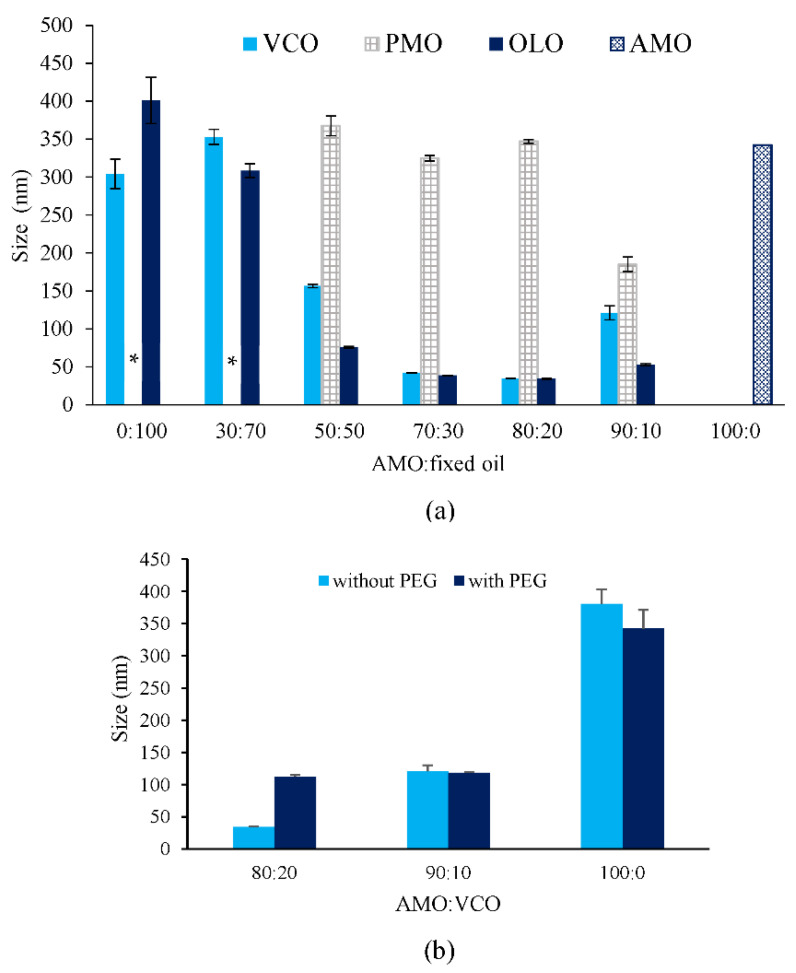
Droplet size of the nanoemulsions prepared from different ratios of AMO:fixed oil and fixed oil type (**a**) and droplet size of AMO:VCO 80:20, 90:10 and 100:0 nanoemulsions with and without PEG 4000 (**b**). * represents cracking. The values are expressed as mean ± SD (*n* = 3).

The size and distribution of the droplet have a significant effect on the permeability of the drug and the stability of the nanoemulsion. A small droplet size results in enhanced thermodynamic stability and drug penetration [20,21]. Figure 1a depicts the droplet size of nanoemulsion formulations containing various types of fixed oil and ratios of AMO to fixed oil. Nanoemulsions prepared from AMO:OLO in ratios ranging from 50:50 to 90:10 and AMO:VCO from 70:30 to 80:20 exhibited droplet sizes that were smaller than 100 nm, whereas nanoemulsions produced from PMO did not produce small-sized nanoemulsions at any of these ratios (physical characteristics of nanoemulsions are shown in Supplementary Data S1). The results were consistent with a previous study suggesting that the type and ratio of AMO to fixed oil has an impact on the formation of nanoemulsions containing essential oils [17].

The effect of chain length and the conformation of fixed oil on the properties of nanoemulsions containing spearmint oil with Cremophor RH40 (P. C. Drug Center, Bangkok, Thailand) as a surfactant were reported by Tubtimsri et al. [17]. Shorter total chain lengths enabled the formation of a smaller droplet size compared with those containing higher total chain lengths. At all ratios, the droplet size of spearmint oil:VCO (trilaurin:C12) was less than that of the PMO system (tripalmitin:C16). Despite the fact that OLO contains more carbon atoms than PMO, it had a smaller droplet size because of a shorter overall projected chain length (triolein = 35.9 Å and tripalmitin = 41.6 Å). It appeared that the shorter part of the fatty acid is favorable for nanoemulsion formation when using polyoxyethylene castor oil derivatives as surfactants (i.e., Kolliphor EL, Cremophor RH40, Sigma-Aldrich, St Louis, MO, USA). This may be the result of alterations in the molecular arrangement of surfactants. Weerapol et al. also revealed a similar influence of the molecular structure of mixed surfactants on the droplet size of nanoemulsions [22]. After incorporating mixed surfactants into SEDDS, Span 80 (containing oleyl side chain) was inserted into the hydrophobic alkyl chain of Kolliphor EL and contributed a repulsive force to increase chain curvature, resulting in a small droplet size. In the present study, VCO and OLO had a shorter total chain length compared with PMO, resulting in a smaller droplet size. In addition, OLO, which contained an unsaturated fatty acid similar to that found in the oleyl side chain of Span 80, may be accordingly docked and pushed into the hydrophobic chain of Kolliphor EL, leading to more curvature of the surfactant and a smaller droplet size compared with that of PMO (long chain saturated fatty acid). Although OLO contained an unsaturated fatty acid, there was no difference in nanoemulsion size compared with VCO (medium chain saturated fatty acid) at a suitable ratio of AMO:fixed oil. Therefore, the total chain length of the fatty acid was the most important factor that affected nanoemulsion droplet size in this study.

The reduction of the ratio between viscosity of the internal to external phase (ηD/ηC) is a strategy to inhibit the Ostwald ripening phenomenon [14]. In the present study, PEG 4000 was selected as a viscosity-inducing agent for the external of the nanoemulsions, whereas the AMO:VCO nanoemulsion was chosen as a representative of the AMO:fixed oil nanoemulsion because of its relatively smaller droplet size. As depicted in Figure 1b, the droplet size of PEG 4000 containing nanoemulsions was not decreased at any AMO:VCO ratio, whereas AMO:VCO 80:20 with PEG 4000 exhibited a larger droplet size compared with that without PEG 4000, although the increased viscosity of the external phase was observed, suggesting that a reduction of the ratio between the viscosity of the internal and external phases could not prevent Ostwald ripening. This may be explained by the increment of interfacial tension between the water and oil from PEG 4000 that resulted in larger droplet formation [23,24], especially in the AMO:VCO 80:20 system.

### 3.2. Oil Droplet Morphology

**Figure 2:**
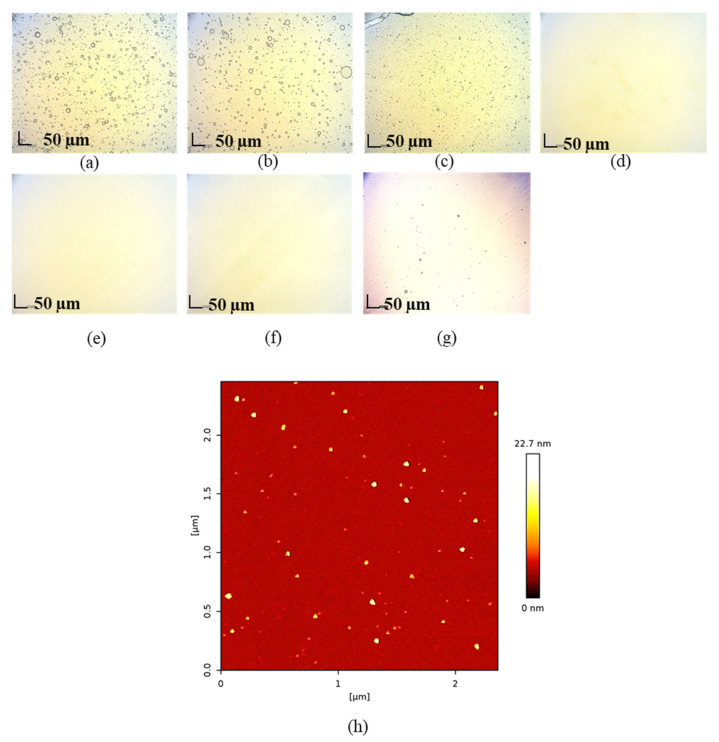
Microscopic images of AMO:VCO 0:100 (**a**), 30:70 (**b**), 50:50 (**c**), 70:30 (**d**), 80:20 (**e**), 90:10 (**f**), 100:0 (**g**) and the AFM image of AMO:VCO 80:20 (**h**).

An optical light microscope or an AFM was used to examine the morphology of oil droplets in a representative AMO:VCO nanoemulsion. As shown in Figure 2, globular droplets with a broad distribution were formed in nanoemulsions with AMO:VCO ratios of 30:70, 50:50, and 0:100. Minuscule droplets smaller than the detection capacity of the optical microscope were observed at 70:30, 80:20, and 90:10 (microscopic images of other systems, PMO and OLO, are shown in Supplementary Data S2). Therefore, the AFM was used to further characterize the submicron size of the formulations, as demonstrated by the AFM image of AMO:VCO 80:20 (Figure 2h), which had the smallest size. The spherical droplets were also observed, confirming the formation of O/W nanoemulsions, even at a droplet size of less than 50 nm.

### 3.3. Stability

**Figure 3:**
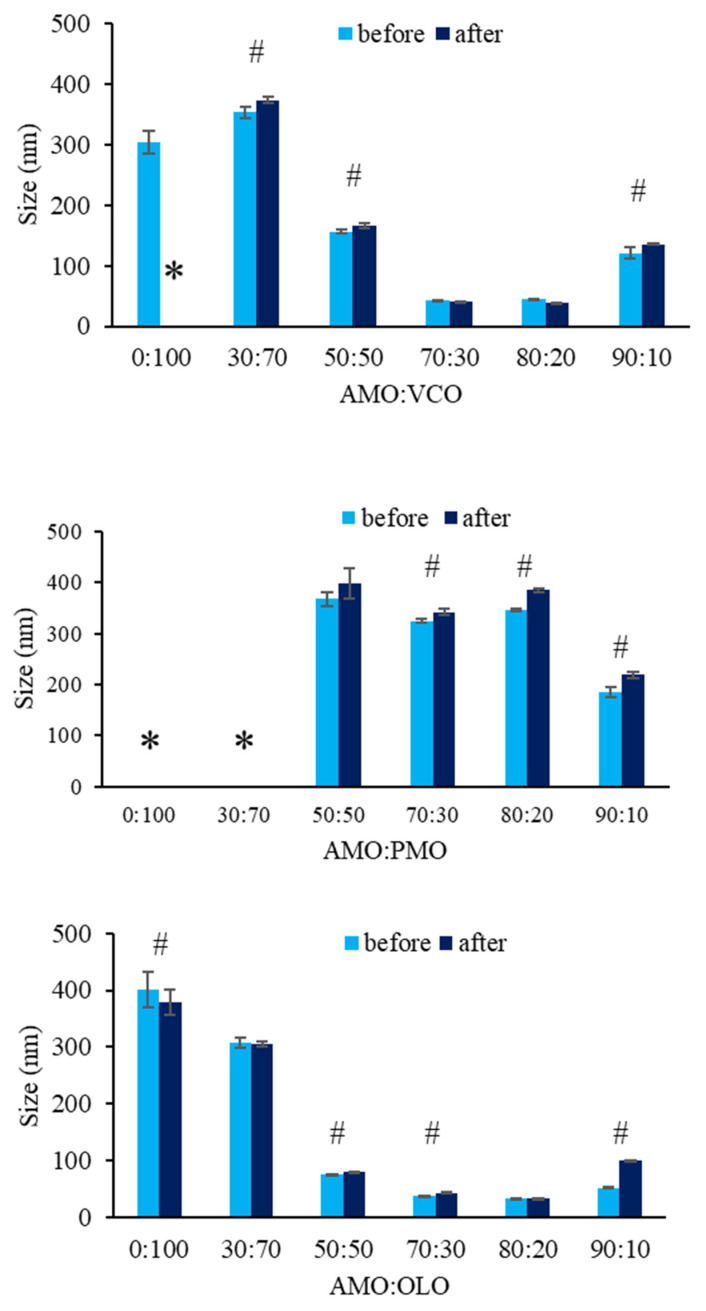
Droplet size of the nanoemulsions prepared from different ratios of AMO:fixed oil and fixed oil type before and after the temperature cycling test, * indicates cracking and # indicates significant difference (*p* < 0.05). The values are expressed as mean ± SD (*n* = 3).

A stability study using temperature cycling was performed to determine the efficacy of the Ostwald ripening inhibitor. AMO:fixed oil nanoemulsions at a ratio of 70:30 and 80:20 prepared from OLO and 80:20 from VCO, were not significantly different with respect to droplet size, indicating that both fixed oils are effective Ostwald ripening inhibitors when used at an acceptable concentration. In contrast to nanoemulsions prepared from PMO, the droplet size increased at all ratios, indicating that PMO was not effective as an Ostwald ripening inhibitor (Figure 3).

Park et al. reported that medium chain triglycerides had more potential as Ostwald ripening inhibitors in orange oil nanoemulsions compared with long chain triglycerides. The Ostwald ripening rate of nanoemulsions was delayed as the mole fraction of orange oil to triglycerides in the oil phase increased [18,25]. In the present study, the MW of trilaurin (main component in VCO) was greater than that of palmitin (main component in PMO), resulting in an increase in the mole fraction of AMO to VCO compared with AMO to PMO. Therefore, it may be possible to explain the higher physical stability of AMO:VCO nanoemulsions. In the case of unsaturated triglyceride, OLO, Tubtimsri et al. concluded that the stability of a nanoemulsion established from spearmint oil (containing monoterpene; carvone) and perilla oil (containing trilinolenin; C18:3) resulted from the molecular interaction among Cremophor RH40, trilinolenin, and carvone, which encouraged surfactant coverage on the surface of the oil droplets [17]. This may explain the stability of nanoemulsions in OLO systems containing chemical compounds comparable to those described in the studies above [17].

### 3.4. ^1^H–^1^H (NOESY) NMR Spectroscopy

**Figure 4:**
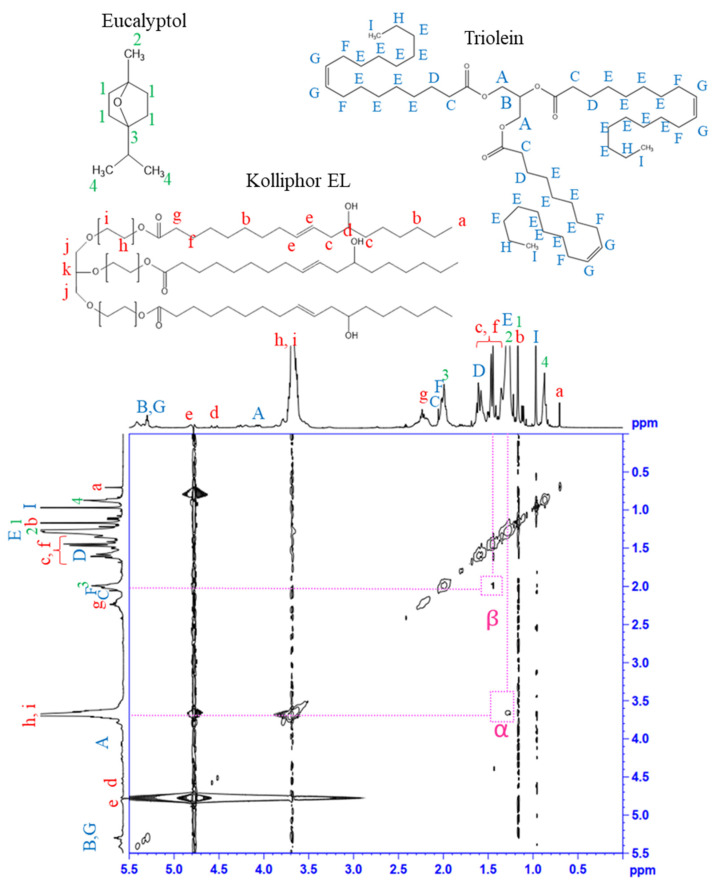
^1^H-^1^H NOESY NMR spectrum of AMO:OLO 80:20 nanoemulsion.

The interaction among molecules was examined using 2D-NOESY NMR, which provides non-bonded proton interactions within 5 Å. As shown in Figure 4, the proton signal and chemical structures of OLO (triolein) and Kolliphor EL are assigned by capital and lowercase letters, respectively. Eucalyptol, the main component of AMO, is represented by a number. The 2D-NOESY spectrum showed the interaction among components as indicated by several cross-peaks. An interaction between the methyl proton from AMO (2) with polyoxyethylene from Kolliphor EL (h, i) and the alkyl chain proton from OLO (E) with polyoxyethylene from Kolliphor EL (h, i) were evident, as indicated by α. Moreover, a cross peak between Kolliphor EL (c, f) and OLO (C, F) was discovered (β). Likewise, the interaction among molecules was also found in the AMO:VCO 80:20 nanoemulsion (Supplementary Data S3). These interactions among the components may be a significant factor for determining the droplet size and stability of nanoemulsions. The AMO and OLO molecules interposed between the polyoxyethylene chains of Kolliphor EL and may increase the curvature of the surfactant, resulting in the creation of small droplets. In addition, the interaction of the components enhanced surfactant coverage at the oil droplet interface, thus increasing the stability of the nanoemulsion.

### 3.5. Ex Vivo Permeability

Although previous studies have demonstrated that nanoemulsions can penetrate cells through the transcellular pathway [26], additional penetration mechanisms should be identified to determine why nanoemulsions are more effective against cancer cells compared with solutions. The TEER measurement is a frequently used tool for assessing the integrity of tight junction dynamics in tissues and epithelial monolayer cell culture models [27]. In the present study, TEER values were used to determine the permeability of nanoemulsions across the paracellular channel. Table 2 compares the TEER values for the nanoemulsion treatment group to those for the AMO solution and PBS. A decrease in TEER value was observed in the nanoemulsion treatment group, whereas the TEER value for the AMO solution treatment group was not significantly different, suggesting that the AMO nanoemulsion also penetrated the cancer cells through a paracellular mechanism, which was not detected in an AMO solution. As a result, the AMO loaded into the nanoemulsion carrier should be more potent compared with the AMO solution.

### 3.6. Anticancer Activity

The 1% (*v*/*v*) 80:20 nanoemulsions prepared from different fixed oils were selected to study the effect of a fixed oil type on the anticancer properties of AMO nanoemulsions using an MTT assay. AMO:OLO exhibited the greatest cytotoxic effect among the fixed oils, with a cytotoxicity value of 99.68 ± 0.56%, followed by AMO:PMO and AMO:VCO (Table 3). The cytotoxicity of all nanoemulsions was significantly different from the base, indicating that the anticancer activity of the nanoemulsions was not only the result of the surfactant, but was also influenced by the carrier and oil phase composition. Additionally, AMO nanoemulsions had lower cytotoxicity on MRC-5 than KON cells, with a cytotoxicity value in the range from 41.18–51.91%, suggesting that AMO nanoemulsions were safe when used to treat oral cancer.

Previous studies demonstrated that the chain length and melting point of fatty acids in fixed oil had an influence on drug permeability, leading to increased treatment efficacy. Regarding straight chain fatty acids, longer acyl side chains promoted penetration into the bilayer of the cell membrane, resulting in impaired membrane integrity and enabling significant drug penetration through the cell membrane [28]. This may be a reasonable explanation for the greater cytotoxic effect of PMO compared with VCO. However, OLO, which contains unsaturated fatty acids, showed no correlation. Muranush et al. demonstrated that there was a relationship between cell permeability and the flexibility of the acyl side chain of fatty acid moieties, which was represented by the melting point. Fatty acids possessing low melting points and high permeability may improve drug penetration compared with those exhibiting high melting points, which results from the enhancement of membrane fluidity [29]. According to our findings, the melting point of OLO is −5.5 °C [30], which is lower than the melting points of PMO (65–67 °C) [31] and VCO (46.5 °C) [32]. As a result, the increased cytotoxicity of OLO compared with PMO and VCO may result from increased cell permeability caused by the low melting point of the fatty acid components. In addition, the cytotoxicity of the nanoemulsion formulation was more pronounced compared with that of their solutions because of the enhanced permeability effect of the nanoemulsions. These results correlated with our permeability study. AMO permeability may be promoted through a paracellular pathway, which is a complementary permeation route in conjunction with the main mechanism (transcellular route). This phenomenon was not found in AMO solutions, which may explain why nanoemulsions are more active than solutions.

**Table 3:** Cytotoxicity in KON cells and MRC-5 cells after treatment with AMO:fixed oil 80:20 nanoemulsions.

Formulations	Cytotoxicity (%)	
	KON	MRC-5
AMO:VCO	67.23 ± 6.06 ^a^	51.91 ± 8.60
AMO:PMO	83.44 ± 2.26 ^a^	45.02 ± 7.30
AMO:OLO	99.68 ± 0.56 ^a^	41.18 ± 12.52
AMO solution	49.27 ± 5.06	-
Base	53.20 ± 6.78	-

The values are expressed as mean ± SD (*n* = 3), ^a^ indicates *p* value < 0.05 when compared with base.

### 3.7. Cell Invasion Analysis

**Figure 5:**
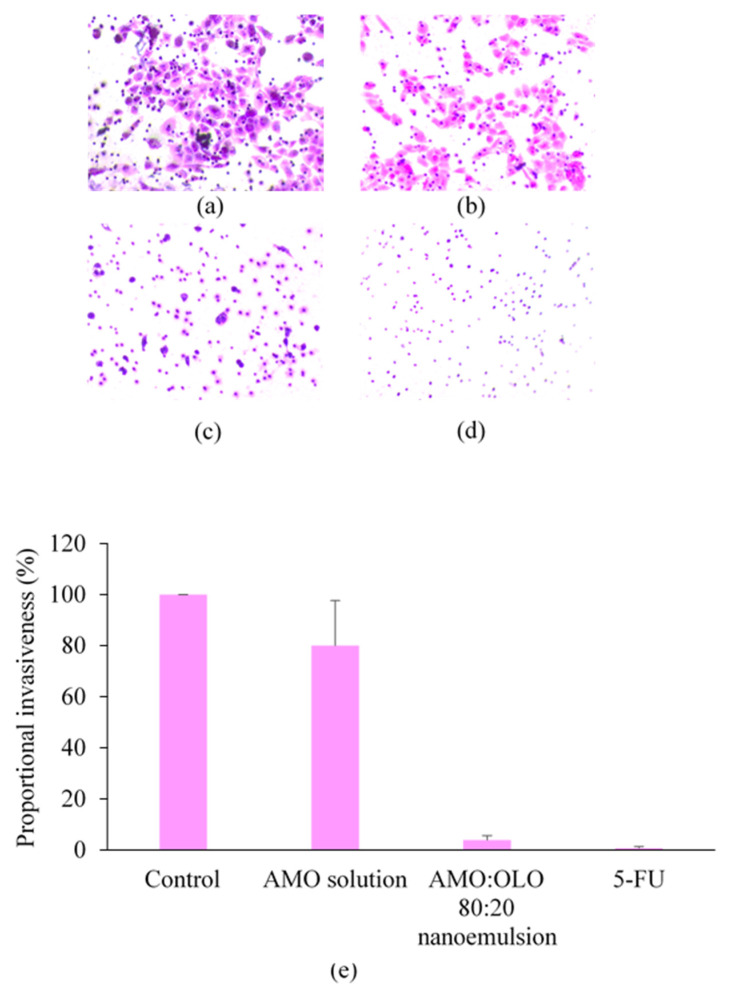
Invasion assay after treatment with control (**a**), AMO solution (at concentrations equivalent to that used for the nanoemulsions) (**b**), AMO:OLO 80:20 nanoemulsion at an IC_60_ concentration (**c**), 30 µg/mL of 5-FU (**d**), and the calculated percentage of proportional invasiveness for each treatment (**e**). The values are expressed as mean ± SD (*n* = 3).

Metastasis is defined as the ability of cancer cells to disseminate and thrive in locations other than their origin, which complicates cancer treatment. The anti-metastatic effect of AMO nanoemulsions was studied by assessing the inhibition of cell invasion, which is a component of cancer metastasis. The AMO:OLO 80:20 nanoemulsion at IC_60_ concentration equaling 0.70% (*v*/*v*) (calculated from the dose–response curve in Supplementary Data S4) was selected as a representative because it was the most potent against oral cancer cell among the fixed oils used. The percentage of proportional invasiveness of the AMO nanoemulsion and 5-FU treatment was markedly decreased compared with AMO solution (Figure 5), suggesting an anti-metastasis effect of the AMO nanoemulsions.

In addition, the AMO nanoemulsion exhibited an anti-clonogenic property, which may promote its anti-metastatic effect (Supplementary Data S5). This may be caused by the antioxidant property of AMO [33], which was also reported for *Illicium verum* essential oils [34]. Similar to the cytotoxicity study described above, the anti-metastatic activity of nanoemulsions may result from the enhanced permeability effect of AMO through the nanoemulsion carrier.

### 3.8. Apoptosis Determination

**Figure 6:**
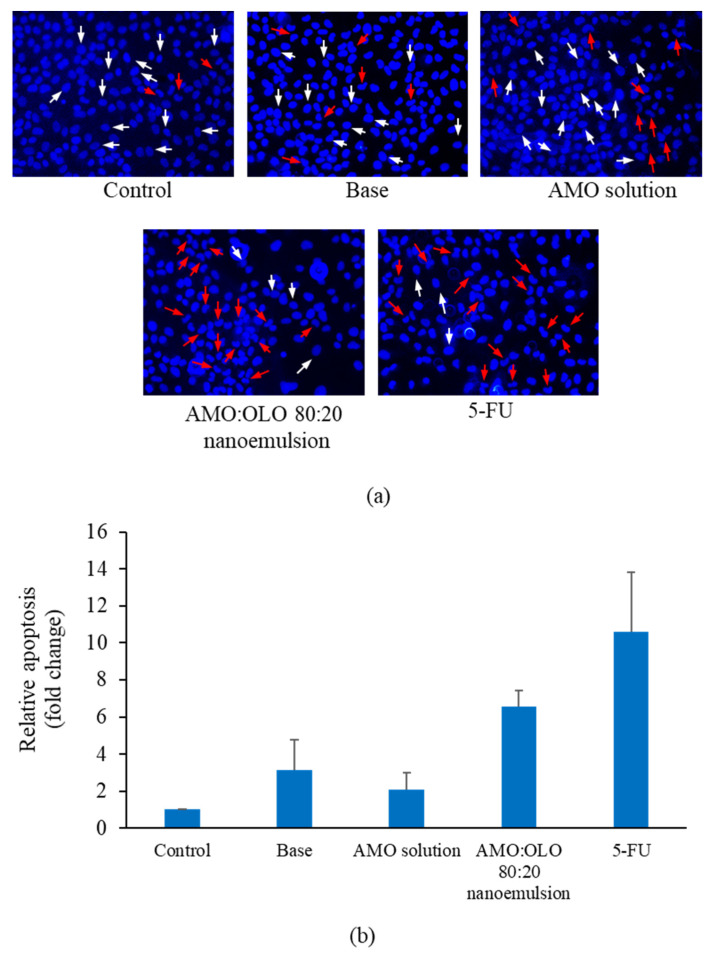
Nuclear fragmentation of KON cells (**a**) and relative apoptosis (**b**) determined by DAPI staining after treatment with control, base (10% *w*/*w* of Kolliphor EL), AMO solution (at concentrations equal to those used in nanoemulsions), IC_60_ of AMO:OLO 80:20 nanoemulsion, and 30 µg/mL of 5-FU. White and red arrows represent normal and apoptotic nuclei, respectively. The values are expressed as mean ± SD (*n* = 3).

Apoptosis is a preferable cell death type for anticancer agents because it is not associated with the inflammatory pathway [35]. To determine the type of cell death generated by the AMO nanoemulsions, nuclear fragmentation, exposure of phosphatidylserine on cell surface and related mRNA expression levels were examined by DAPI staining, annexin V-FITC/DAPI staining and qPCR, respectively. The AMO:OLO 80:20 nanoemulsion was selected as a representative nanoemulsion because it was the most effective against oral cancer cells compared with the other fixed oils and their solutions. An increase in apoptotic cells was observed following treatment with AMO:OLO 80:20 nanoemulsion, which were 6.57 ± 0.86 times greater compared with the control and comparable to 5-FU treatment. In addition, the nanoemulsion was more effective than the solution or base (Figure 6).

**Figure 7:**
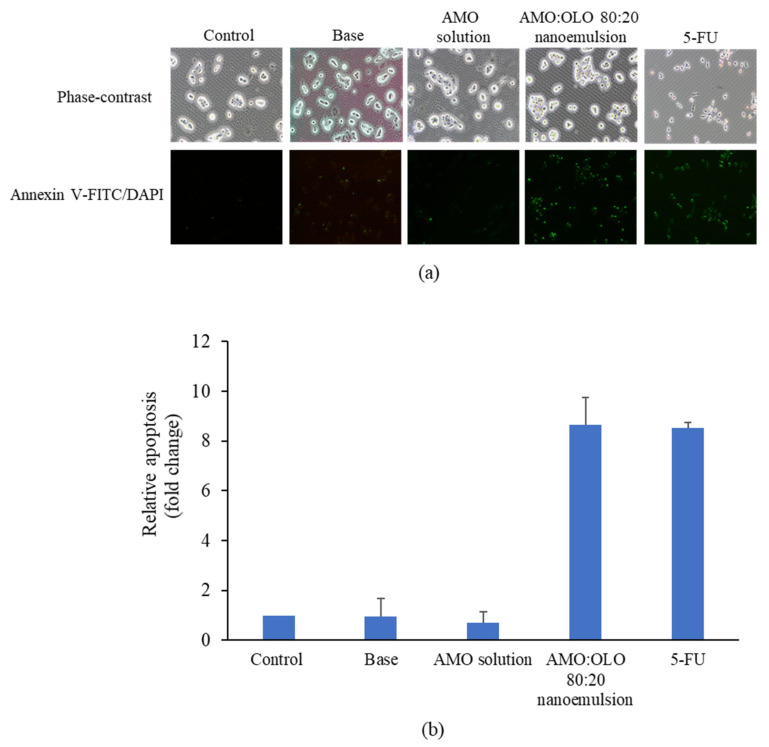
Phase-contrast and fluorescence (annexin V-FITC and DAPI staining) images of KON cells (**a**) and relative apoptosis (**b**) determined by annexin V-FITC/DAPI staining after induction with base (10% *w*/*w* of Kolliphor EL), AMO solution (at concentrations equivalent to that used in the nanoemulsions), AMO:OLO 80:20 nanoemulsion at an IC_60_ concentration, and 30 µg/mL of 5-FU. The values are expressed as mean ± SD (*n* = 3).

To distinguish apoptosis from necrosis, annexin V-FITC and DAPI were used to stain KON cells after induction with samples. Apoptosis was positive to annexin V-FITC while late apoptotic or necrosis was stained with both of annexin V-FITC and DAPI due to loss of integrity of the cell membrane and nuclear membrane. As depicted in Figure 7, the apoptotic cells were 8.67 ± 1.08 times and 8.52 ± 0.23 times greater in number after induction with AMO:OLO 80:20 nanoemulsion and 5-FU, respectively, compared with control. The apoptosis of the base and AMO solution treatment group was not clearly different from the control. In addition, since there was no disruption to the nuclear membrane, and DAPI could not penetrate the nucleus. DAPI-positive labeling was not clearly observed.

**Figure 8:**
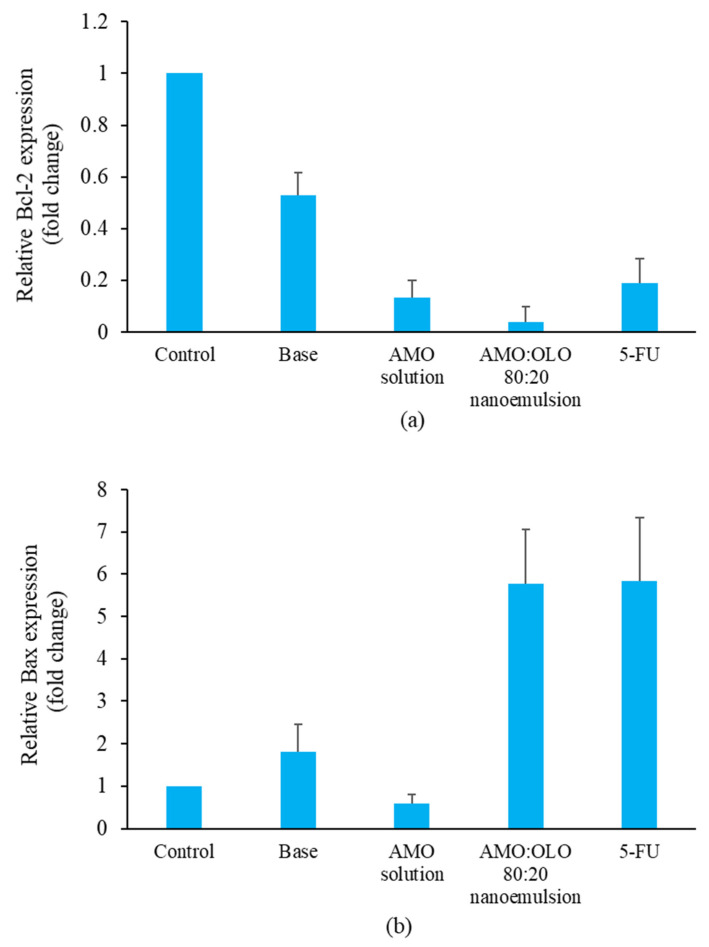
Relative Bcl-2 expression levels by qPCR (**a**) and relative Bax expression levels using qPCR (**b**) after induction with base (10% *w*/*w* of Kolliphor EL), AMO solution (at concentrations equivalent to that used in the nanoemulsions), AMO:OLO 80:20 nanoemulsion at an IC_60_ concentration and 30 µg/mL of 5-FU. The values are shown as mean ± SD (*n* = 3).

The expression levels of Bcl-2 (anti-apoptotic gene) and Bax (proapoptotic gene) were significantly altered in the AMO:OLO 80:20 treatment group. Bcl-2 levels were markedly reduced 0.04 ± 0.06 times, whereas Bax levels were significantly increased to 5.77 ± 1.30 times in comparison with control. The result was similar to that of 5-FU treatment, which could reduce Bcl-2 to 0.19 ± 0.10 times and increase Bax to 5.83 ± 1.50 times. Base and AMO solution group were able to slightly alter Bcl-2 and Bax levels, which were 0.53 ± 0.09 times and 0.13 ± 0.07 times for Bcl-2 and 1.80 ± 0.65 and 0.59 ± 0.21 for Bax, (Figure 8). A previous study has shown that AMO exhibits antioxidant activity [33], which increases P53 expression, promotes Bax expression, and inhibits Bcl-2 expression [36,37]. Either an increase in Bax or a decrease in Bcl-2 results in a leak in the mitochondrial membrane, which is attributed to cytochrome c release. Cytochrome c can activate caspases, which induced cell death by apoptosis. Complementary results from nuclear alterations, exposure of phosphatidylserine on the cell surface and mRNA expression levels suggest that nanoemulsion formulations of AMO can inhibit oral cancer cells by intrinsic apoptosis induction.

## 4. Conclusions

We successfully developed nanoemulsion formulations of AMO by incorporating fixed oils as Ostwald ripening inhibitors. The suitable ratio of AMO to fixed oil was 80:20, except for PMO, which exhibited nanosized droplets and acceptable stability. This phenomenon was associated with the structure of the main component in fixed oil. The small droplet size and acceptable stability may be the result of an interaction among molecules, which could promote the curvature of Kolliphor EL, as indicated by 2D-NOESY NMR. Among the nanoemulsions, AMO:OLO 80:20 exhibited the highest activity against oral cancer cells, which involved intrinsic apoptosis induction and also showed anti-metastasis activity. Additionally, the AMO nanoemulsions demonstrated lower toxicity on normal cells (MRC-5) as compared to KON cancer cells. These findings indicate that the AMO nanoemulsion formulation has the potential for the treatment of oral cancer and may lead to strategies for the fabrication of stable nanoemulsions containing various volatile oils.

## Figures and Tables

**Table 1:** Composition of the AMO nanoemulsion.

Formulations	Fixed Oil Type (% *w*/*w*)			AMO (% *w*/*w*)	Kolliphor EL (% *w*/*w*)	PEG 4000 (% *w*/*w*)	Water (% *w*/*w*)
	VCO	PMO	OLO				
AMO:VCO							
100:0	-	-	-	10.0	10.0	-	80.0
90:10	1.0	-	-	9.0	10.0	-	80.0
80:20	2.0	-	-	8.0	10.0	-	80.0
70:30	3.0	-	-	7.0	10.0	-	80.0
50:50	5.0	-	-	5.0	10.0	-	80.0
30:70	7.0	-	-	3.0	10.0	-	80.0
0:100	10.0	-	-	-	10.0	-	80.0
AMO:PMO							
90:10	-	1.0	-	9.0	10.0	-	80.0
80:20	-	2.0	-	8.0	10.0	-	80.0
70:30	-	3.0	-	7.0	10.0	-	80.0
50:50	-	5.0	-	5.0	10.0	-	80.0
30:70	-	7.0	-	3.0	10.0	-	80.0
0:100	-	10.0	-	-	10.0	-	80.0
AMO:OLO							
90:10	-	-	1.0	9.0	10.0	-	80.0
80:20	-	-	2.0	8.0	10.0	-	80.0
70:30	-	-	3.0	7.0	10.0	-	80.0
50:50	-	-	5.0	5.0	10.0	-	80.0
30:70	-	-	7.0	3.0	10.0	-	80.0
0:100	-	-	10.0	-	10.0	-	80.0
100:0 with 5% PEG 4000	-	-	-	10.0	10.0	5.0	75.0
90:10 with 5% PEG 4000	-	-	1.0	9.0	10.0	5.0	75.0
80:20 with 5% PEG 4000	-	-	2.0	8.0	10.0	5.0	75.0

**Table 2:** TEER values across porcine buccal before and after treatment with AMO:OLO 80:20 nanoemulsion, AMO solution and PBS.

Samples	Before	After
AMO:OLO 80:20 nanoemulsion	269.84 ± 56.56	103.96 ± 12.12 ^a^
AMO solution	263.00 ± 17.55	236.60 ± 55.09
PBS	333.35 ± 69.29	290.71 ± 67.07

The values are expressed as mean ± SD (*n* = 3), ^a^ indicates *p* value < 0.05 when compared with before treatment.

## Supplementary Materials

The following supporting information can be downloaded at: https://www.mdpi.com/article/10.3390/pharmaceutics14050938/s1, Supplementary Data S1: Physical characteristics of nanoemulsions prepared from different types and amounts of fixed oils. Supplementary Data S2: Microscopic images of AMO:PMO 0:100 (a), 30:70 (b), 50:50 (c), 70:30 (d), 80:20 (e), 90:10 (f). Microscopic images of AMO:OLO 0:100 (a), 30:70 (b), 50:50 (c), 70:30 (d), 80:20 (e), 90:10 (f). Supplementary Data S3: ^1^H–^1^H NOESY spectra of AMO:VCO 80:20. Supplementary Data S4: Dose response curve of KON cells after treatment with AMO:OLO 80:20 nanoemusion. Supplementary Data S5: Colony morphology after treatment with control (a), AMO solution (at concentrations equal to those used in nanoemulsions) (b), IC_60_ of AMO:OLO 80:20 nanoemulsion (c) and 30 µg/mL of 5-FU (d) for 15 min.
