# Supplementary material for: Impact of Fixed Oil on Ostwald Ripening of Anti-Oral Cancer Nanoemulsions Loaded with Amomum kravanh Essential Oil
Source: Pharmaceutics. 2022 Apr 26;14(5):938. doi: 10.3390/pharmaceutics14050938 (PMC9146979; doi:10.3390/pharmaceutics14050938)
Supplement: Supplementary file 1 [file pharmaceutics-14-00938-s001.zip › pharmaceutics-1676867-supplementary.pdf]

pharmaceutics

MDPI

Supplementary Materials: Impact of Fixed Oil on Ostwald Ripening of Anti-Oral Cancer Nanoemulsions Loaded with Amomum Kravanh Essential Oil

Yotsanan Weerapol, Suwisit Manmuan, Nattaya Chaothanaphat, Siriporn Okonogi, Chutima Limmatvapirat, Sontaya Limmatvapirat and Sukannika Tubtimsri

Supplementary Data S1

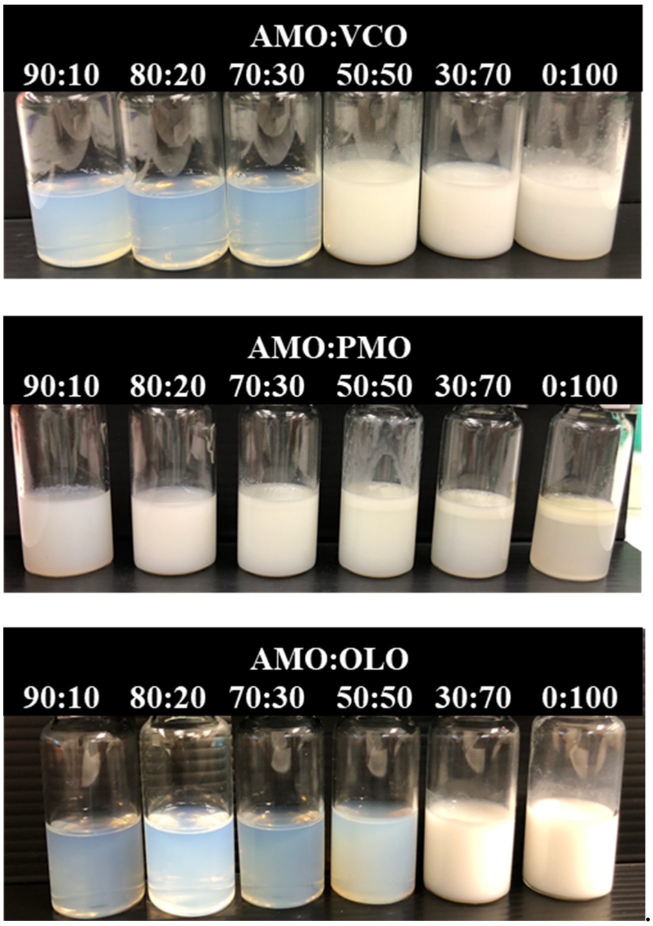

Physical characteristics of nanoemulsions when prepared from different types and amounts of fixed oils.

Supplementary Data S2

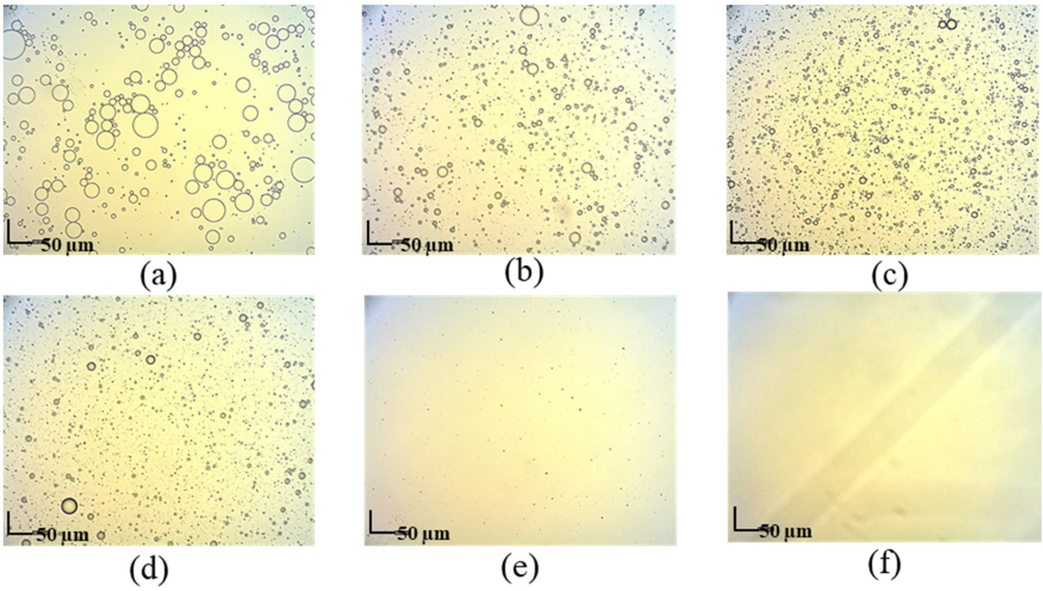

Microscopic pictures of AMO:PMO 0:100 (a), 30:70 (b), 50:50 (c), 70:30 (d), 80:20 (e), 90:10 (f).

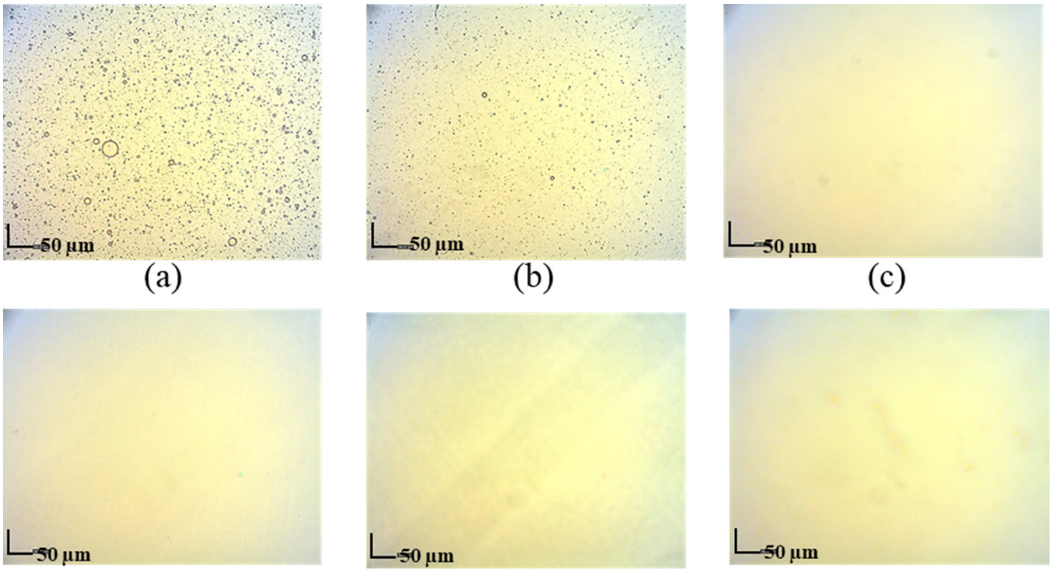

Microscopic pictures of AMO:OLO 0:100 (a), 30:70 (b), 50:50 (c), 70:30 (d), 80:20 (e), 90:10 (f).

Supplementary Data S3

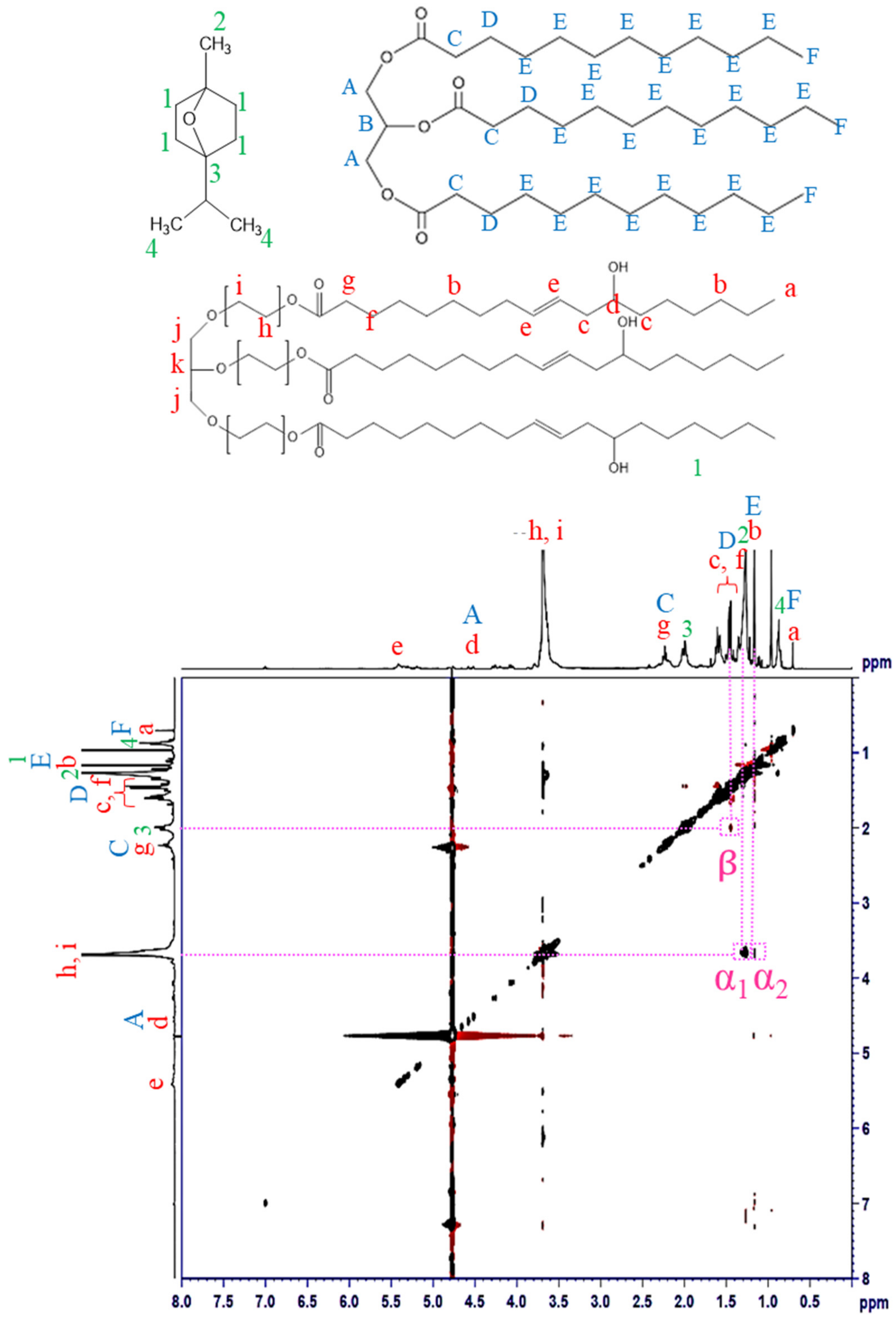

^{1}H–^{1}H NOESY spectra of AMO:VCO 80:20

Supplementary Data S4

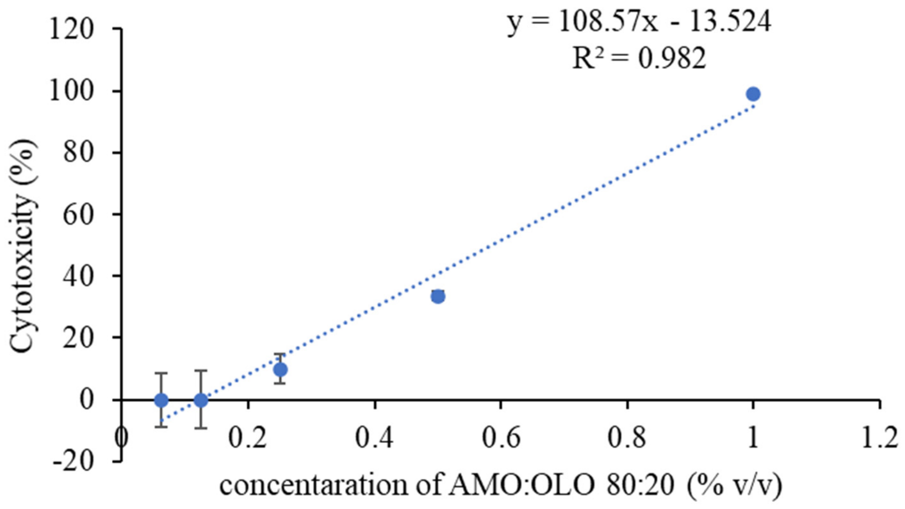

Dose response curve of KON cells after treatment with AMO:OLO 80:20 nanoemusion

Supplementary Data S5

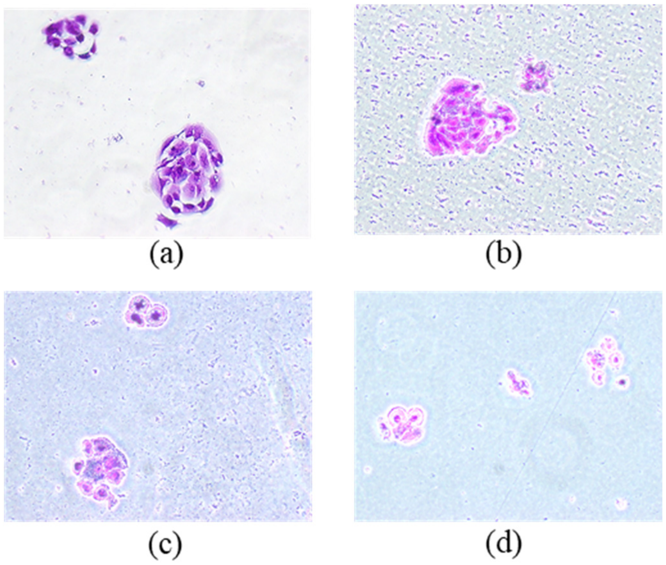

Colony morphology after treatment with control (a), AMO solution (at concentrations equal to those used in nanoemulsions) (b), IC_{60} of AMO:OLO 80:20 nanoemulsion (c) and 30 µg/ml of 5-FU (d) for 15 min.
